# Robotic Conversion of Sleeve Gastrectomy to Gastric Bypass in a Patient With Situs Inversus Totalis: A Case Report

**DOI:** 10.7759/cureus.93971

**Published:** 2025-10-06

**Authors:** Naofal da Silva, Khaled Omar, Laura Barbosa, Hope M Cherian, Michelle Gallas, Anthony M Gonzalez

**Affiliations:** 1 General Surgery, AdventHealth, Tampa, USA; 2 Surgery, Baptist Health South Florida, Miami, USA; 3 Humanities, Health, and Society, Herbert Wertheim College of Medicine, Miami, USA; 4 Center for Research, Baptist Health South Florida, Miami, USA

**Keywords:** case report, gastric bypass, robotic gastric bypass, roux-en-y, situs inversus totalis, sleeve gastrectomy

## Abstract

Bariatric surgeries are increasingly commonplace in treating morbid obesity and the associated comorbidities. Situs inversus totalis (SIT) represents a rare anatomical deviation in which the thoracic and abdominal viscera are in mirror-image positions compared with normal anatomy. The paucity of literature on surgical outcomes of this condition underscores the need for this report. Understanding the preoperative workup and unique operative technical challenges will help surgeons better prepare for such rare cases. We present a case of a 54-year-old female with known SIT, morbid obesity, obstructive sleep apnea, and cardiac arrhythmias with an automatic implantable cardioverter defibrillator (AICD) in place. The patient presented with complaints of regurgitation and weight regain seven years after laparoscopic sleeve gastrectomy at another facility. After appropriate preoperative workup, the patient was offered conversion to gastric bypass. We proceeded with a robotic conversion of sleeve gastrectomy to gastric bypass with no intraoperative or postoperative events. At the one-month follow-up visit, the patient tolerated her diet and was compliant with medications and vitamins. This report represents the first documented case of robotic sleeve conversion to gastric bypass in a patient with SIT. Robotic surgery is useful in improving surgical precision and ergonomics in complex cases and highlights its feasibility in such rare cases. We conclude that robotic bariatric surgery is safe in patients with SIT, provided that all preoperative workup is completed and necessary anatomical precautions are taken during the procedure.

## Introduction

Situs inversus totalis (SIT) is a rare congenital condition characterized by a complete mirror-image reversal of the thoracic and abdominal organs [1]. This anatomical anomaly presents unique challenges in clinical practice due to the unfamiliar orientation of internal structures. Although individuals with SIT typically lead normal lives without physiological impairment, the altered anatomy can complicate diagnostic processes and surgical interventions [2]. Medical imaging and physical examinations may require careful interpretation, as clinicians must adapt to the reversed positioning of organs such as the heart, liver, stomach, and intestines.

In surgical contexts, particularly those involving the abdomen, the presence of SIT demands meticulous preoperative planning and intraoperative navigation. Surgeons must modify their standard approaches to accommodate the reversed anatomy, which can otherwise lead to disorientation or inadvertent injury [3]. This complexity is amplified in minimally invasive procedures, where visual cues are critical for orientation. Therefore, comprehensive preoperative imaging and detailed anatomical mapping are essential to minimize operative risks and improve outcomes.

Bariatric surgery has gained widespread acceptance as an effective treatment for morbid obesity and its related comorbidities, including diabetes, hypertension, and sleep apnea. More than 260,000 cases are performed annually according to the American Society for Metabolic and Bariatric Surgery (ASMBS) [4]. As the number of bariatric procedures continues to rise globally, more patients with uncommon anatomical variations, such as SIT, are likely to present for surgical management. While laparoscopic sleeve gastrectomy (LSG) and Roux-en-Y gastric bypass (RYGB) remain the most frequently performed bariatric operations, reports of these procedures in patients with SIT are scarce. Furthermore, conversion surgeries, such as transitioning from sleeve gastrectomy (SG) to RYGB, are even less commonly documented in this population.

The surgical management of patients with SIT undergoing bariatric procedures requires adaptation in operative technique, careful intraoperative orientation, and thorough preoperative assessment. Sharing such cases contributes valuable insights to the surgical community, highlighting potential pitfalls, technical modifications, and strategies for safe and effective management. This case report aims to describe the clinical presentation, surgical approach, and postoperative course of a patient with SIT undergoing conversion bariatric surgery, underscoring the importance of tailored surgical planning in this unique subset of patients. This case report has been prepared in line with the SCARE (Surgical CAse REport) checklist [5].

## Case presentation

We present the case of a 54-year-old morbidly obese female with SIT who presented for an RYGB after an LSG seven years earlier. At the time of evaluation, her BMI was 47 kg/m². The patient had a past medical history of SIT, idiopathic ventricular fibrillation requiring automatic implantable cardioverter-defibrillator (AICD) placement, and obstructive sleep apnea treated with continuous positive airway pressure (CPAP) therapy. The patient’s surgical history included LSG and partial nephrectomy.

**Figure 1:**
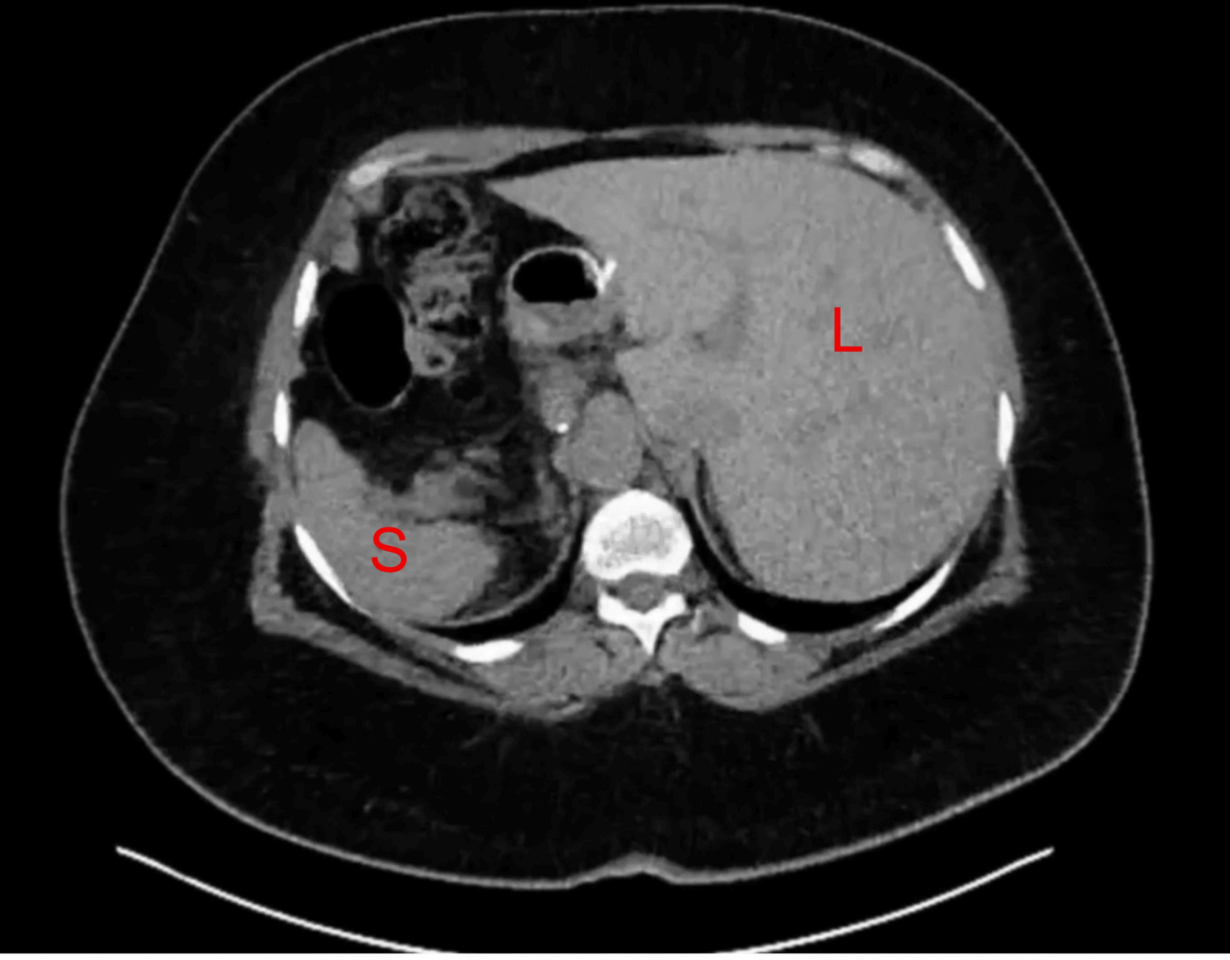
Abdominal CT scan shows organs in opposite anatomical positions Liver (L) seen on the left side of the body and spleen (S) on the right side of the body.

**Figure 2:**
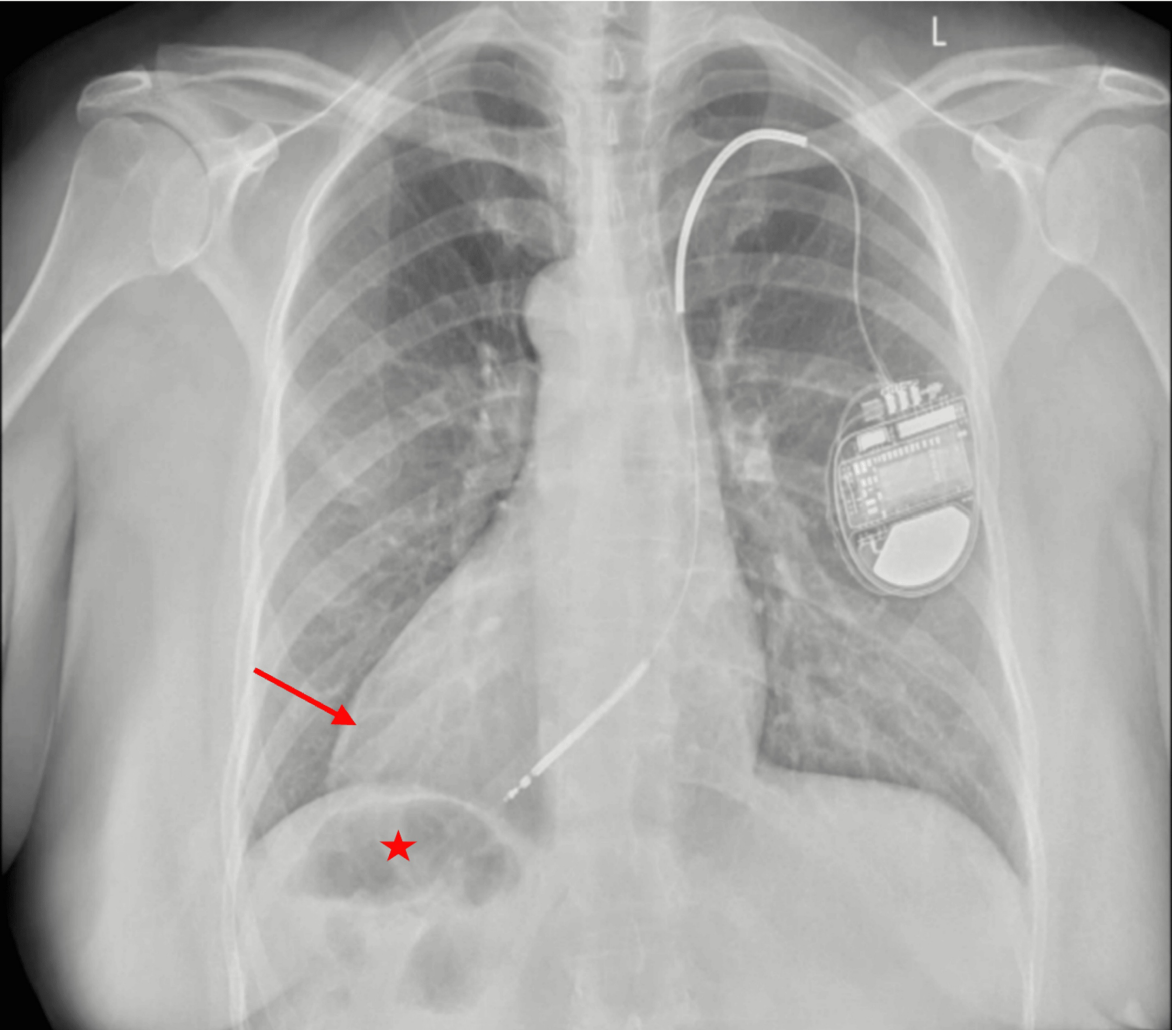
Chest X-ray The cardiac apex (arrow) is directed toward the right hemithorax, consistent with dextrocardia, and the stomach (star) is located in the right upper quadrant.

The patient presented with regurgitation and weight regain after LSG. Imaging studies confirmed SIT (Figure 1) and demonstrated an AICD implant in situ (Figure 2). Preoperative endoscopy revealed a dilated sleeve with a spiral effect and multiple areas of narrowing in the mid-sleeve and near the incisura, with the Z-line located at 41 cm.

As for the surgical procedure, the patient was positioned supine, and general anesthesia was induced. Ports were placed in a mirror-image fashion compared with standard laparoscopic RYGB procedures [6]. In standard laparoscopic RYGB procedures, ports are typically placed predominantly on the left side of the abdomen to provide optimal access and angles for instrument maneuvering. This usually includes a periumbilical or left-sided camera port, with additional working ports placed in the left upper and middle quadrants, and occasionally one on the right side to facilitate dissection and stapling. In contrast, ports were placed in a mirror-image fashion compared with this standard configuration.

**Figure 3:**
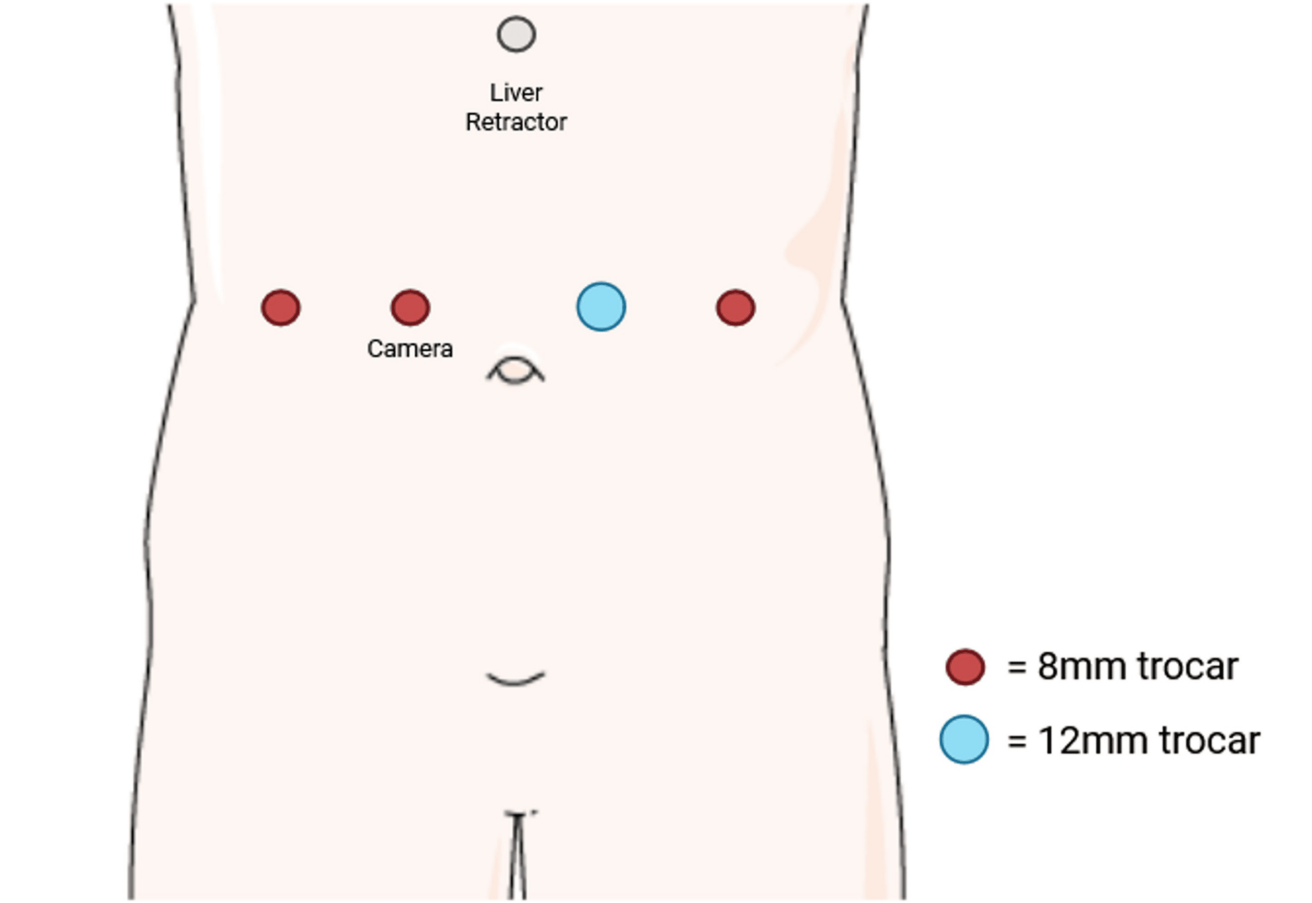
Trocar placement Three 8-mm trocars were placed in total: two on the right side of the abdomen and one on the left lateral side. Additionally, one 12-mm trocar was placed in the left middle quadrant. Image adapted from Servier Medical Art (https://smart.servier.com/), licensed under CC BY 4.0 (https://creativecommons.org/licenses/by/4.0/).

A periumbilical right-sided incision was made, and abdominal entry was achieved using a 5-mm Optiview trocar. The abdomen was insufflated to 15 mmHg with carbon dioxide. Three 8-mm trocars were placed in total: two on the right side of the abdomen and one on the left lateral side. One 12-mm trocar was placed in the left middle quadrant (Figure 3). A Nathanson liver retractor was placed in the subxiphoid position. Situs inversus was confirmed, with the patient’s liver and gallbladder on the left side of the body cavity and the ligament of Treitz (LOT) on the right side. There was evidence of a previous SG, with adhesions on the right lateral aspect of the sleeve. The inferior mesenteric vein was visualized on the right lateral aspect of the LOT. The bowel was run to the 50-cm point. A stitch was placed 50 cm distal to the LOT. The robot was docked at the patient’s left side, and the LOT was reidentified. The small bowel was run to the 50-cm point where the stitch was located. The small bowel was transected just proximal to this stitch with a linear stapler, leaving the stitch on the alimentary limb. The alimentary limb was measured to be 150 cm in length. An enteroenterostomy was created with the biliopancreatic limb at the 150-cm point. A linear stapler was used to create the jejunojejunostomy, and the small bowel opening was closed with a robotic stapler. The small bowel was sutured to prevent angulation. Although SIT posed a potential challenge, anatomical landmarks were accurately identified at multiple points during the procedure. The omentum was divided vertically to allow the alimentary limb to reach the gastric pouch without tension. The lesser omentum was opened. Adhesions on the right lateral aspect of the previous LSG were taken down with the vessel sealer. A 40-French bougie, inserted earlier, was retracted into the esophagus. The stomach was transected horizontally just distal to the left gastric artery with a linear stapler. The bougie was then passed back down to occupy the entirety of the new gastric pouch. The small bowel alimentary limb was brought in antecolic antegastric fashion and sutured to the right lateral aspect of the small gastric pouch.

Enterotomies were created in the pouch and small bowel, and a robotic linear stapler was used to create a small common enterotomy. The opening was closed with a 3-0 PDS barbed suture in a two-layer running fashion. Following completion of the anastomosis, an upper endoscopy was performed, which demonstrated a patent and adequate anastomosis. The gastric pouch was also deemed adequate, and when insufflated with air while submerged in water, no leakage was observed, as evidenced by the absence of bubbles. The pouch and small bowel were suctioned, and the endoscope was removed. Hemostasis was ensured. The anatomy was reinspected. The gastric pouch was traced along the alimentary limb to the level of the jejunojejunostomy. The biliopancreatic limb was also followed, with clear delineation of the postoperative anatomy.

The patient had an uncomplicated postoperative recovery and was discharged on postoperative day 1. Early follow-up revealed the resolution of reflux symptoms and satisfactory weight loss.

## Discussion

In recent years, obesity has become one of the most prevalent diseases worldwide. Bariatric surgery has proven to be an effective solution for treating morbid obesity, offering long-lasting results [7]. These interventions modify the gastrointestinal tract through distinct surgical approaches to promote weight loss and metabolic improvement [8].

Although RYGB was long considered the gold standard for permanent weight loss surgery, SG gained popularity due to its higher survival rate, shorter operative time, and similar complication rates [9,10]. SG, however, is more frequently associated with complications such as gastroesophageal reflux disease (GERD), weight regain, and insufficient weight loss compared with RYGB, which may lead to the need for revisional surgery [11,12].

A literature review reported 39 patients with SIT who had undergone bariatric surgery [13]. From the articles reviewed, only two revisions were identified: one in 2012, a laparoscopic vertical SG after open gastric banding [14], and the other a revision of an SG to RYGB in a patient with established SIT [15]. Our report serves as the second documented case of a conversion of SG to RYGB in a patient with SIT, while also being the first to describe the operation performed through a robotic approach.

SIT presents a rare but significant anatomical variation that demands thorough planning from the bariatric surgeon. Mirror-image port placement and orientation modifications are crucial for safe and effective procedures. Robotic gastric bypass conversion from SG can achieve favorable outcomes in patients with SIT when performed by an experienced surgical team.

This case illustrates the distinct technical challenges encountered during complex, rare procedures. A robotic approach not only aided in addressing the anatomical difficulties associated with organ inversion but also highlighted its usefulness in improving surgical precision and ergonomics in this complex case [16]. Although only a few similar case reports are available in the literature, the findings of this case support the feasibility and safety of using robotic technology for bariatric procedures in patients with significant anatomical variations. These findings also emphasize the critical role of tailored surgical strategies and meticulous preoperative preparation in developing adaptive surgical approaches.

## Conclusions

This case highlights the successful management of a complex bariatric surgical conversion in a patient with SIT, a rare and challenging anatomical variation. It emphasizes the critical importance of adapting surgical techniques to accommodate individual anatomical variations, ensuring both the safety and effectiveness of the procedure. The use of a robotic approach in this case demonstrated its value in enhancing precision, overcoming technical challenges, and improving overall surgical ergonomics. Furthermore, this case underscores the necessity of meticulous preoperative planning, including detailed imaging and team collaboration, to address the complexities associated with such unique conditions. Ultimately, this experience reinforces the potential of advanced surgical technologies in managing difficult cases and optimizing patient outcomes.
